# Identification of genomic alterations and associated transcriptomic profiling reveal the prognostic significance of MMP14 and PKM2 in patients with pancreatic cancer

**DOI:** 10.18632/aging.103958

**Published:** 2020-09-17

**Authors:** Haiwei Wang, Xinrui Wang, Liangpu Xu, Yingying Lin, Ji Zhang, Hua Cao

**Affiliations:** 1 Medical Research Center, Fujian Maternity and Child Health Hospital, Affiliated Hospital of Fujian Medical University, Fuzhou, Fujian, China; 2 Key Laboratory of Technical Evaluation of Fertility Regulation for Non-human Primate, National Health and Family Planning Commission, Fuzhou, Fujian, China; 3 Department of Neurosurgery, Renji Hospital, Shanghai Jiao Tong University School of Medicine, Shanghai, China; 4 State Key Laboratory for Medical Genomics, Shanghai Institute of Hematology, Ruijin Hospital, Shanghai Jiao Tong University School of Medicine, Shanghai, China

**Keywords:** pancreatic cancer, KRAS mutation, TP53 mutation, CDKN2A deletion, MMP14

## Abstract

Pancreatic cancer is characterized by multiple genomic alterations, including KRAS mutations, TP53 mutations and CDKN2A deletion. However, the prognostic relevance of those genomic alterations and associated transcriptomic profiling in pancreatic cancer are unclear. Integrated analysis of The Cancer Genome Atlas (TCGA) datasets revealed that KRAS mutation, TP53 mutation and CDKN2A deletion were all bad prognostic factors in pancreatic cancer. And KRAS mutation, TP53 mutation and CDKN2A deletion were coordinated and co-occurred in pancreatic cancer. Transcriptomic analysis showed that MMP14 and PKM2 were both up-regulated by KRAS mutation, TP53 mutation or CDKN2A deletion. Also, MMP14 and PKM2 were both associated with unfavorable outcomes in pancreatic cancer. Compared with normal tissues, MMP14 and PKM2 were up-regulated in pancreatic cancer tissues. Moreover, MMP14 and PKM2 were highly expressed in high grade of pancreatic cancer. Furthermore, MMP14 and PKM2 were correlated with each other, and the combination of MMP14 and PKM2 could be used as better prognostic markers than MMP14 or PKM2 alone. At last, the high expression and bad prognostic effects of MMP14 and PKM2 in pancreatic cancer were validated using tissue microarray. Overall, the genomic alterations and associated transcriptomic profiling analysis suggested new prognostic makers of MMP14 and PKM2 in pancreatic cancer.

## INTRODUCTION

Pancreatic cancer is a highly aggressive disease and one of leading cause of cancer related mortality [1, 2]. Most pancreatic cancer patients are diagnosed at advanced stages due to nonspecific symptoms. The treatment options of pancreatic cancer are limited [3–5]. Moreover, most of pancreatic cancer patients are resistant to current therapies [6, 7], contributing to the worst prognosis of pancreatic cancer. The 5-year overall survival rate of pancreatic cancer is only 5% -8% [8, 9]. Although, mutational landscape [10], gene expression [11], microRNA signature [12] and lncRNA expression profiling [13] are used as biomarkers for patients with pancreatic cancer, effective new diagnostic and prognostic biomarkers are badly needed [14].

As the collection of genomic data, the biological behaviors of drive mutations in pancreatic cancer are extensively studied. The first whole-exome sequencing study suggests that pancreatic cancer is characterized by KRAS mutation, TP53 mutation, CDKN2A deletion and SMAD alteration [15]. After that, whole-genome sequencing [16], integrated genomic and transcriptomc analysis [17], including efforts from TCGA network [18] validate the KRAS, TP53 mutations and CDKN2A, SMAD copy number alterations in pancreatic cancer. KRAS mutation is required for the initiation and maintenance of malignant state of pancreatic cells by regulation of metabolism and senescence [19–21]. KRAS mutation also cooperates with TP53 mutation [22] or SMAD4 alteration [23] to promote the metastasis of pancreatic cancer. Moreover, pancreatic cancer patients with KRAS mutation, TP53 mutation, CDKN2A deletion and SMAD alteration tend to have worse clinical outcomes [24–26]. However, some long-term survivors of pancreatic cancer patients are not determined by the genetic mutations [27]. So, the synergism of KRAS mutation, TP53 mutation, CDKN2A deletion and SMAD alteration in the prognosis of pancreatic cancer still need to be illustrated. Furthermore, the prognostic effects of genomic alterations associated transcriptomic profiling in pancreatic cancer are unknown.

In the present study, using large cohorts of pancreatic cancer patients derived from TCGA datasets and Gene Expression Omnibus (GEO) datasets, the prognostic significance of KRAS mutation, TP53 mutation, CDKN2A deletion and SMAD alteration was determined. Also transcriptomic profiling associated with KRAS mutation, TP53 mutation and CDKN2A deletion and their prognostic effects were identified and validated in pancreatic cancer.

## RESULTS

### Prognostic relevance of genomic alterations in patients with pancreatic cancer

**Figure 1:**
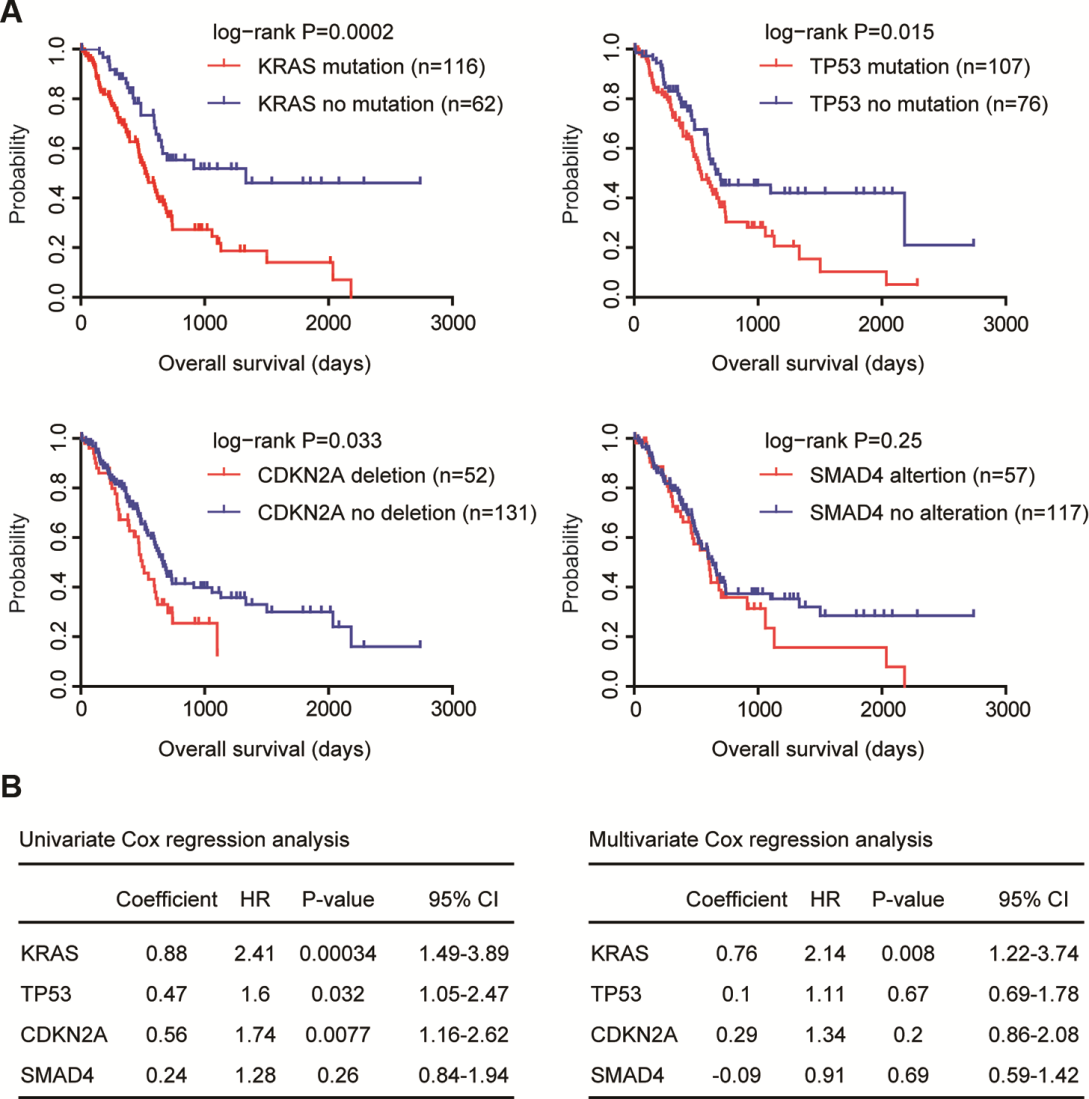
**Prognostic relevance of genomic alterations in patients with pancreatic cancer.** (**A**) Kaplan-Meier plots demonstrated the prognostic effects of KRAS, TP53, CDKN2A and SMAD4 alterations in patients with pancreatic cancer in TCGA PAAD datasets. The log-rank test was used to determine the different overall survival between patients with (red) or without (blue) genomic alterations. (**B**) Univariate and multivariate cox regression were used to test the prognostic significance of KRAS, TP53, CDKN2A and SMAD4 alterations in patients with pancreatic cancer in TCGA PAAD datasets. The log-rank test was used to determine the overall survival P-value. HR, hazard ratio; CI, confidence interval.

Pancreatic cancer is characterized with KRAS and TP53 mutation, CDKN2A deletion and SMAD4 alteration. First, we determined the prognosis of those genomic alterations using TCGA Pancreatic adenocarcinoma (PAAD) datasets. We found that KRAS mutation, TP53 mutation and CDKN2A deletion were all associated with the clinical overall survival of patients with pancreatic cancer (Figure 1A). Patients with KRAS mutation, TP53 mutation or CDKN2A deletion demonstrated worse prognosis compared with patients without KRAS mutation, TP53 mutation or CDKN2A deletion (Figure 1A). Moreover, compared with TP53 mutation or CDKN2A deletion, KRAS mutation was a more significant prognostic factor (Figure 1A). However, there was no different clinical overall survival between pancreatic cancer patients with or without SMAD4 alterations (Figure 1A).

Similar conclusions were derived from univariate cox regression analysis. KRAS mutation, TP53 mutation and CDKN2A deletion were all prognostic factors in patients with pancreatic cancer in TCGA datasets (Figure 1B), while, SMAD4 alteration was not associated with the clinical outcomes of pancreatic cancer patients (Figure 1B).

### Coordination and co-occurrence of the genomic alterations in patients with pancreatic cancer

Using multivariate cox regression analysis, we determined the association of KRAS mutation, TP53 mutation and CDKN2A deletion in the prediction of overall survival in pancreatic cancer patients. We found that KRAS mutation was an independent prognostic factor (Figure 1B). However, TP53 mutation and CDKN2A deletion were interconnected and were not independent prognostic factors (Figure 1B).

**Figure 2:**
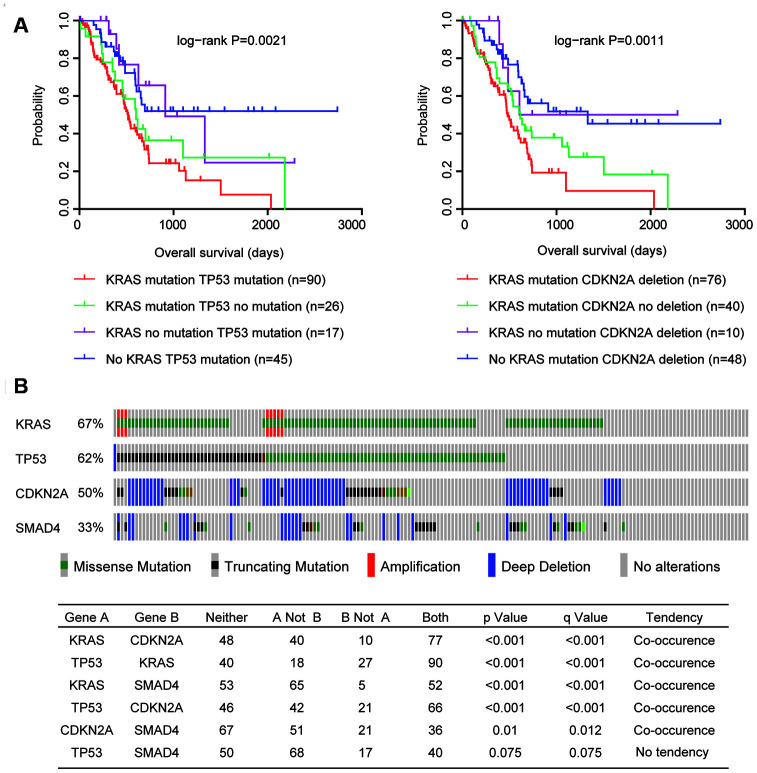
**Coordination and co-occurrence of the genomic alterations in patients with pancreatic cancer.** (**A**) Kaplan-Meier plots demonstrated the different overall survival of pancreatic cancer patients with different genomic alterations. P values were generated from Log-rank test. (**B**) Oncoprint demonstrated the co-occurrence of KRAS, TP53, CDKN2A and SMAD4 alterations in patients with pancreatic cancer derived from TCGA PAAD datasets. Each line represented one patient.

Next, we tested the combination of KRAS mutation and TP53 mutation in determining the overall survival of patients with pancreatic cancer. Patients with both KRAS mutation and TP53 mutation had worst prognosis than patients with KRAS mutation or TP53 mutation, or without mutations (Figure 2A). Moreover, patients with both KRAS mutation and CDKN2A deletion also had worst prognosis than patients with KRAS mutation or CDKN2A deletion, or without alterations (Figure 2A). Results derived from multivariate cox regression and Kaplan-Meier analysis suggested the coordination of KRAS mutation, TP53 mutation and CDKN2A deletion in the predication of overall survival of pancreatic cancer patients.

Furthermore, genetically, KRAS mutation, TP53 mutation and CDKN2A deletion were also connected. Among the 175 pancreatic cancer patients in TCGA PAAD datasets, 117 patients were with KRAS mutation, 108 patients were with TP53 mutation and 87 patients were with CDKN2A deletion (Figure 2B). Interestingly, 90 pancreatic cancer patients (51%) were with both KRAS and TP53 mutations, 77 pancreatic cancer patients (44%) were with both KRAS mutation and CDKN2A deletion, and 66 pancreatic cancer patients (37%) were with both TP53 mutation and CDKN2 deletion (Figure 2B). Statistic analysis showed that the co-occurrence of KRAS mutation, TP53 mutation and CDKN2A deletion was significant (Figure 2B). However, SMAD4 alteration was not significantly co-occurred with TP53 mutation or CDKN2A deletion (Figure 2B).

### Identification of genomic alterations associated transcriptomic profiling

**Figure 3:**
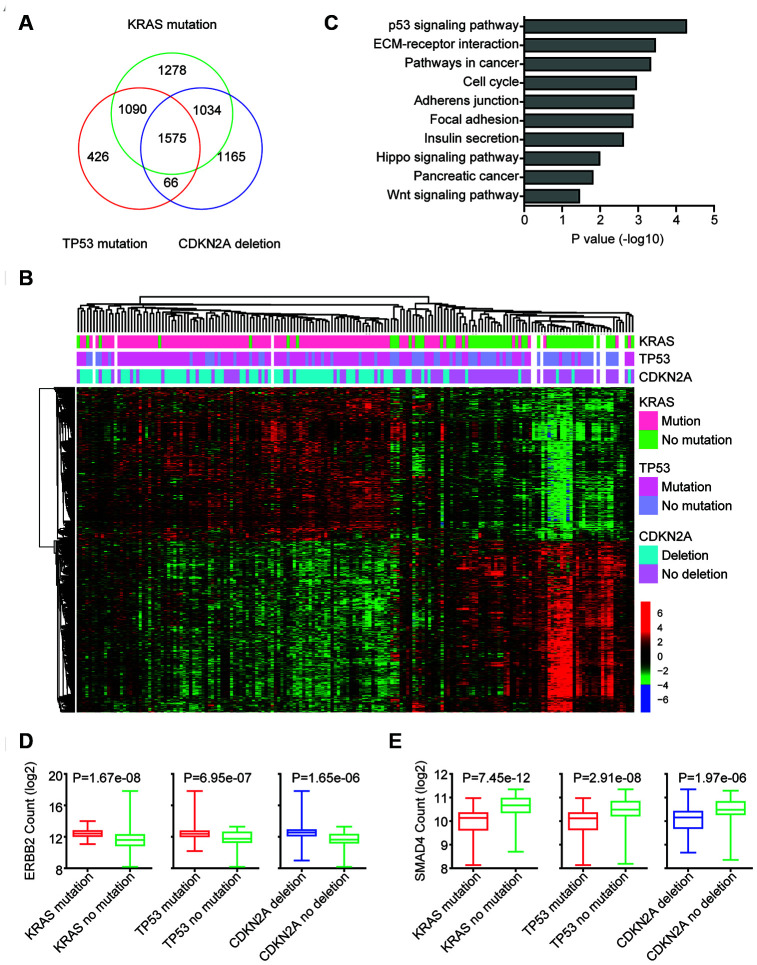
**Identification of genomic alterations associated transcriptomic profiling.** (**A**) Venn diagram depicted the number of commonly regulated genes by KRAS mutation, TP53 mutation and CDKN2A deletion in TCGA PAAD datasets. (**B**) Unsupervised clustering heatmap demonstrated the commonly regulated genes by KRAS mutation, TP53 mutation and CDKN2A deletion in TCGA PAAD datasets. Up-regulated (red), down-regulated (green) and unchanged (black) genes were delineated. (**C**) Functional pathway enrichment analysis of KRAS mutation, TP53 mutation and CDKN2A deletion commonly regulated genes using DAVID. The most significantly enriched pathways were shown. (**D**) Box plots showed the ERBB2 expression levels (log2 normalization count) in TCGA pancreatic cancer patients with or without genomic alterations. P values were performed using Student’s t test. (**E**) Box plots showed the SMAD4 expression levels (log2 normalization count) in TCGA pancreatic cancer patients with or without genomic alterations.

The genomic alterations may influent the expression levels of hundred genes to promote the development of pancreatic cancer. Next, the genes regulated by KRAS mutation, TP53 mutation or CDKN2A deletion were identified. We found that 4799 genes were differentially expressed in pancreatic cancer patients with or without KRAS mutation. 3157 genes were differentially expressed in pancreatic cancer patients with or without TP53 mutation. And 3740 genes were regulated by CKDN2A deletion (Figure 3A). Among all the differentially expressed genes, 1575 genes were commonly regulated by KRAS mutation, TP53 mutation and CDKN2A deletion (Figure 3A). And those genes classified the pancreatic cancer patients into two different clusters with different genomic KRAS, TP53 and CDKN2A alterations (Figure 3B).

We further determined the enriched signaling pathways associated with KRAS mutation, TP53 mutation and CDKN2A deletion. Consistent with the TP53 mutation in pancreatic cancer, we found that TP53 signaling pathway was most significantly enriched (Figure 3C). We also found that cell cycle, pathways in cancer, Hippo signaling pathway and Wnt signaling pathway were associated with KRAS mutation, TP53 mutation or CDKN2A deletion in pancreatic cancer (Figure 3C).

Some pancreatic cancer patients are with ERBB2 amplification [16, 18]. We found that ERBB2 expression was also up regulated by KRAS, TP53 and CDKN2A alterations. In pancreatic cancer patients with KRAS mutation, TP53 mutation or CDKN2A deletion, the expression levels of ERBB2 were relatively higher (Figure 3D). Although, SMAD4 was not genetically co-occurred with TP53 and CDKN2A, the expression levels of SMAD4 were also relatively higher in pancreatic cancer patients with KRAS mutation, TP53 mutation or CDKN2A deletion (Figure 3E).

### MMP14 and PKM2 are regulated by KRAS mutation, TP53 mutation and CDKN2A deletion and associated with the prognosis of patients with pancreatic cancer

**Figure 4:**
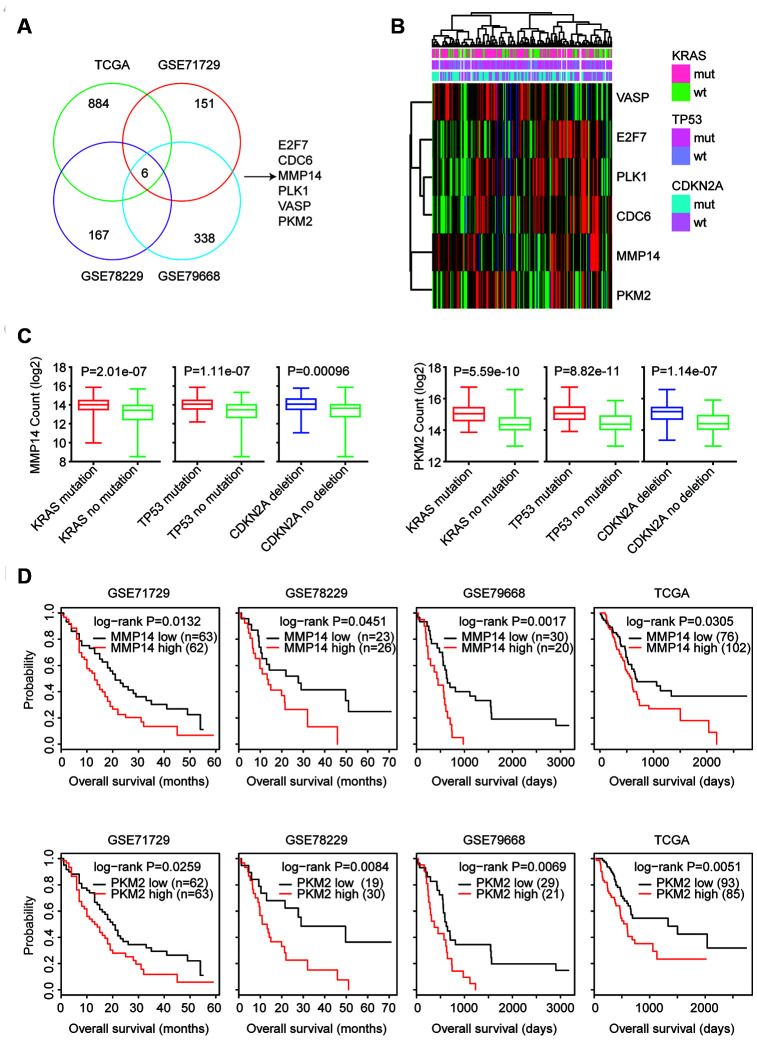
**MMP14 and PKM2 are regulated by KRAS mutation, TP53 mutation and CDKN2A deletion and associated with the prognosis of patients with pancreatic cancer.** (**A**) Venn diagram depicted that six genes E2F7, CDC6, MMP14, PLK1, VASP and PKM2 were associated with the prognosis of patients with pancreatic cancer in TCGA, GSE71729, GSE78229 and GSE79668 datasets. (**B**) Unsupervised clustering heatmap demonstrated the expression levels of E2F7, CDC6, MMP14, PLK1, VASP and PKM2 in TCGA PAAD datasets. (**C**) Box plots showed the MMP14 and PKM2 expression levels (log2 normalization count) in TCGA pancreatic cancer patients with or without genomic alterations. P values were performed using Student’s t test. (**D**) Kaplan-Meier survival analysis was used to compare the overall survival of MMP14 or PKM2 highly expressed patients (red) with MMP14 or PKM2 lowly expressed patients (black) in TCGA, GSE71729, GSE78229 and GSE79668 datasets. P values were generated from Log-rank test.

Next, we determined the prognostic relevance of the 1575 commonly regulated genes by KRAS mutation, TP53 mutation and CDKN2A deletion. Kaplan-Meier survival analysis revealed that 884 genes (56%) were significantly associated with the clinical overall survival of patients with pancreatic cancer derived from TCGA PAAD datasets (Figure 4A). We used another three GEO datasets GSE71729, GSE78229 and GSE79668 to further validate the prognostic relevance of the KRAS mutation, TP53 mutation and CDKN2A deletion commonly regulated genes. 151 genes out of the 1575 genes were demonstrated prognostic effects in GSE71729 dataset. 167 genes in GSE78229 and 338 genes in GSE79668 were also associated with the prognosis of patients with pancreatic cancer (Fig4. A). Interesting, we found six genes E2F7, CDC6, MMP14, PLK1, VASP and PKM2 were significantly correlated with the prognosis of patients with pancreatic cancer in TCGA, GSE71729, GSE78229 and GSE79668 datasets (Figure 4A).

E2F7, CDC6, MMP14, PLK1, VASP and PKM2 were up-regulated in patients with KRAS, TP53 or CDKN2A alterations (Figure 4B). Moreover, MMP14 and PKM2 were clustering with each other as demonstrated in the heatmaps (Figure 4B). Expression levels of MMP14 and PKM2 were further illustrated in the box plots (Figure 4C). Compared with patients without KRAS, TP53 or CDKN2A alterations, MMP14 and PKM2 were highly expressed in pancreatic cancer patients with KRAS, TP53 or CDKN2A alterations (Figure 4C). Moreover, the higher expression levels of MMP14 and PKM2 were correlated with the worse prognosis in patients with pancreatic cancer in TCGA, GSE71729, GSE78229 and GSE79668 datasets (Figure 4D).

### Validation of the prognosis of MMP14 and PKM2 in three GEO datasets

**Figure 5:**
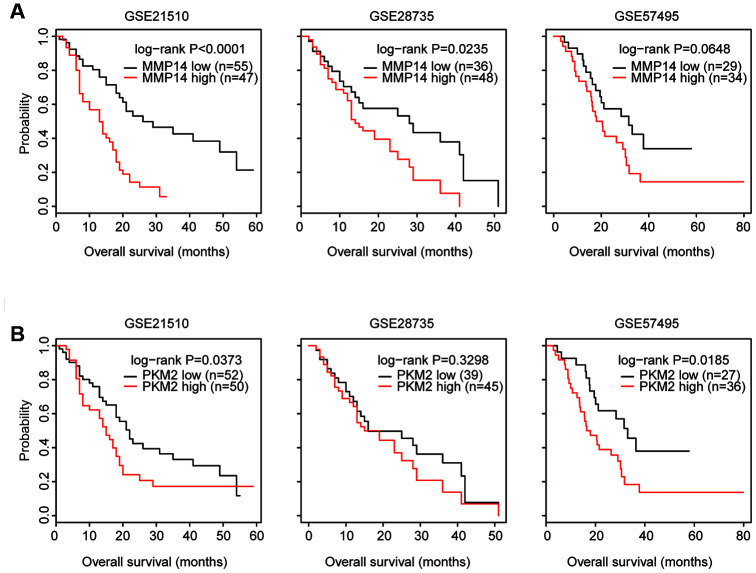
**Validation of the prognosis of MMP14 and PKM2 in three GEO datasets.** (**A**) Kaplan-Meier survival analysis was used to compare the overall survival of MMP14 highly expressed patients (red) with MMP14 lowly expressed patients (black) in GSE21250, GSE28735 and GSE57495 datasets. P values were generated from Log-rank test. (**B**) Kaplan-Meier survival analysis was used to compare the overall survival of PKM2 highly expressed patients (red) with PKM2 lowly expressed patients (black) in GSE21250, GSE28735 and GSE57495 datasets.

Additionally, the prognostic effects of MMP14 and PKM2 were further validated using GSE21520, GSE28735 and GSE57495 datasets. In GSE21510 and GSE28735 datasets, the higher expression level of MMP14 was associated with worse prognosis in patients with pancreatic cancer (Figure 5A). Although less significantly, patients with higher expression level of MMP14 was related to bad clinical outcomes in GSE57495 dataset (Figure 5A).

In GSE21520 and GSE57495 datasets, higher expression level of PKM2 was also associated with worse prognosis in patients with pancreatic cancer (Figure 5B). However, there was no prognostic effect of PKM2 in pancreatic cancer patients in GSE28735 dataset (Figure 5B). Moreover, other genes E2F7, CDC6, PLK1 and VASP showed litter or no prognostic significance in GSE21520, GSE28735 and GSE57495 datasets. Those results highlighted that MMP14 and PKM2 were important prognostic markers in patients with pancreatic cancer.

### Expression levels of MMP14 and PKM2 in normal and malignant pancreatic tissues

**Figure 6:**
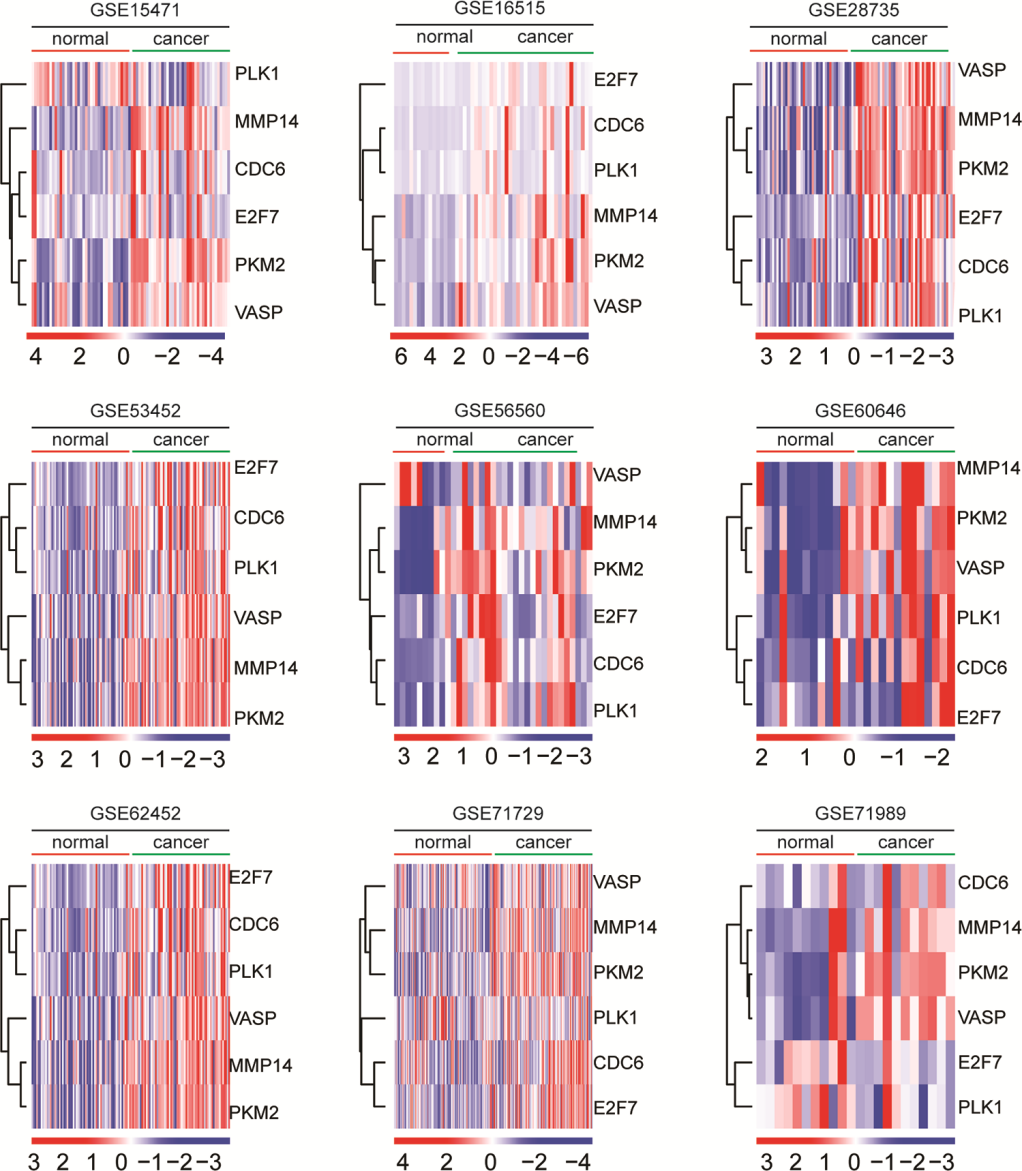
**Expression levels of MMP14 and PKM2 in normal and malignant pancreatic tissues.** Heatmaps demonstrated the expression levels of E2F7, CDC6, MMP14, PLK1, VASP and PKM2 in normal and malignant pancreatic tissues in GSE15471, GSE16515, GSE28735, GSE53452, GSE56560, GSE60646, GSE62452, GSE71729 and GSE71989 datasets. Up-regulated (red) and down-regulated (blue) genes were delineated.

The expression levels of E2F7, CDC6, MMP14, PLK1, VASP and PKM2 in normal and malignant pancreatic tissues were investigated in GSE15471, GSE16515, GSE28735, GSE53452, GSE56560, GSE60646, GSE62452, GSE71729 and GSE71989 datasets. As illustrated in the heatmaps, MMP14 and PKM2 were up-regulated in malignant pancreatic tissues in all nine datasets (Figure 6). Moreover, MMP14 and PKM2 were closely clustered into a small sub-group (Figure 6). The other four genes E2F7, CDC6, PLK1and VASP were also up-regulated in malignant pancreatic tissues (Figure 6).

### Expression levels of MMP14 and PKM2 in different subtypes of patients with pancreatic cancer.

**Figure 7:**
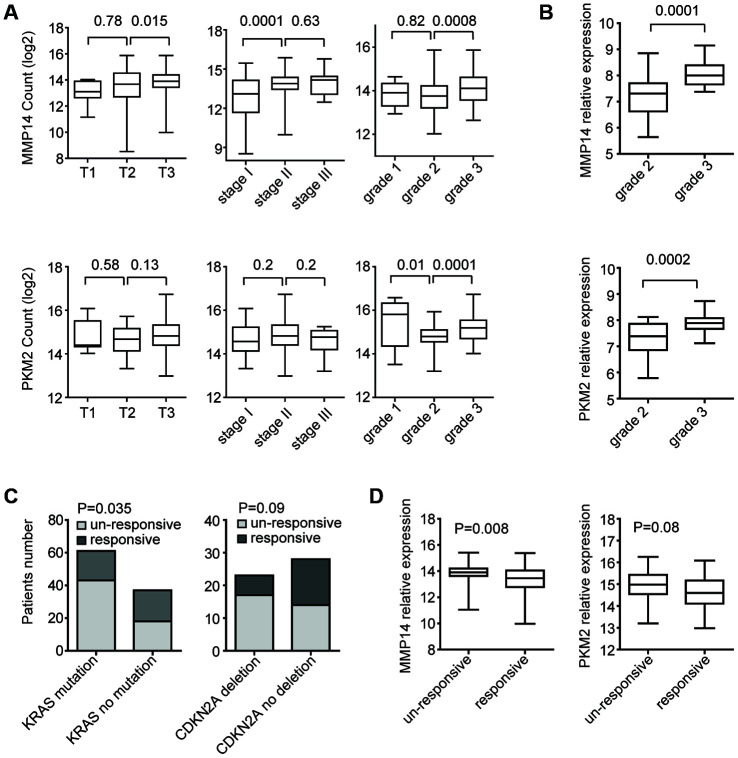
**Expression levels of MMP14 and PKM2 in different subtypes of patients with pancreatic cancer.** (**A**) Box plots demonstrated the expression levels of MMP14 and PKM2 in different subtypes of patients with pancreatic cancer in TCGA datasets. P values were determined using Student’s t test. (**B**) Box plots demonstrated the expression levels of MMP14 and PKM2 in different grades of patients with pancreatic cancer in GSE78829 dataset. (**C**) Contingency graphs showed the number of patients with KRAS or CDKN2A alterations in different clinical responsiveness. P values were determined by Chi-square test. (**D**) Box plots demonstrated the expression levels of MMP14 and PKM2 in treatment responsive or un-responsive patients with pancreatic cancer.

Pancreatic cancer is a heterogeneous disease. We then assessed the expression levels of MMP14 and PKM2 in pancreatic cancer patients with different subtypes. Compared with T2 stage, the expression levels of MMP14 was relatively higher in patients with T3 stage (Figure 7A). Also, MMP14 was highly expressed in stage II and grade 3 pancreatic cancer patients (Figure 7A). However, there were no different expression levels of PKM2 in different stages of patients with pancreatic cancer. Only, compared with grade 2, the expression levels of PKM2 were relatively higher in patients with grade 3 (Figure 7A). Compared with grade 2, the higher expression levels of MMP14 and PKM2 in grade 3 subtype of pancreatic cancer patients were also observed in GSE78829 dataset (Figure 7B).

We also tested whether KRAS mutation, TP53 mutation and CDKN2A deletion were associated with the therapy responsiveness in patients with pancreatic cancer. In TCGA PAAD datasets, 70% pancreatic cancer patients with KRAS mutations were un-responsive to cancer therapy, while, 49% patients without KRAS mutations were un-responsive to cancer therapy (Figure 7C). Statistically, KRAS mutation was significantly associated with the therapy responsiveness (Figure 7C). Similarly, pancreatic cancer patients with CDKN2A deletion were prone to become therapy un-responsive (Figure 7C). Corresponding to the higher un-responsiveness of pancreatic cancer patients with KRAS mutation or CDKN2A deletion, we found that MMP14 and PKM2 were highly expressed in drug un-responsive patients (Figure 7D).

### Combined prognostic significance of MMP14 and PKM2 in patients with pancreatic cancer

**Figure 8:**
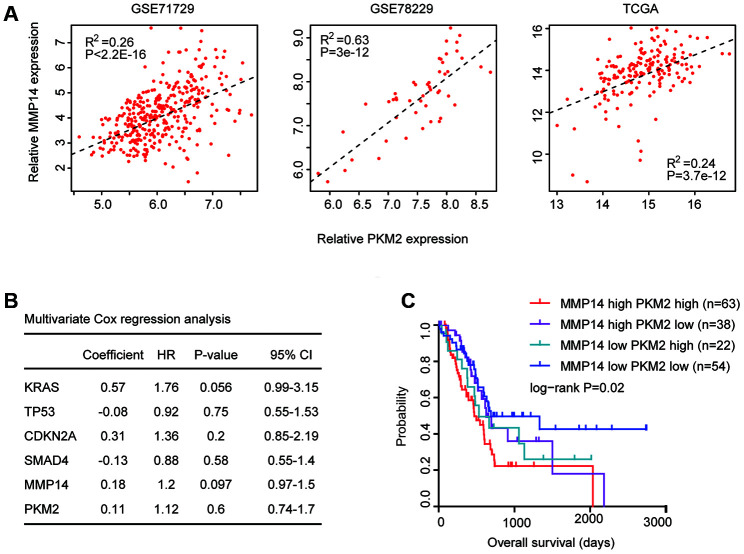
**Combined prognostic significance of MMP14 and PKM2 in patients with pancreatic cancer.** (**A**) Spearman correlation of MMP14 and PKM2 expression levels in GSE71729, GSE78229 and TCGA datasets. (**B**) Multivariate cox regression was used to test the prognostic significance of KRAS, TP53, CDKN2A and SMAD4 alterations and MMP14 and PKM2 expression in patients with pancreatic cancer in TCGA datasets. The log-rank test was used to determine the overall survival P-value. (**C**) Kaplan-Meier plotters demonstrated the different overall survival of pancreatic cancer patients with high expression levels of MMP14 and PKM2 and pancreatic cancer patients with low expression levels of MMP14 and PKM2 in TCGA dataset. Log-rank test was used to determine the P values.

Next, we determined the connections of MMP14 and PKM2 in patients with pancreatic cancer. First, Spearman correlation demonstrated positive correlations of MMP14 and PKM2 in GSE71729, GSE78229 and TCGA datasets (Figure 8A). Patients with high expression levels of MMP14 were also with high expression of PKM2. Second, using multivariate cox regression analysis, we determined the association of MMP14 and PKM2 in overall survival prediction. We found that, MMP14 and PKM2 were not independent prognostic factors in TCGA PAAD datasets (Figure 8B). We thought that the combination of MMP14 with PKM2 could be used as better prognostic markers in patients with pancreatic cancer. Based on the average expression levels of MMP14 and PKM2, pancreatic cancer patients were divided into MMP14 and PKM2 high expression group or MMP14 and PKM2 low expression group. We found that patients with both high MMP14 and PKM2 expression levels were with lowest overall survival in patients with pancreatic cancer (Figure 8C). Patients with the both low expression levels of MMP14 and PKM2 had better clinical overall survival in TCGA datasets (Figure 8C). Those results suggested the combined prognostic significance of MMP14 and PKM2 in patients with pancreatic cancer.

### Validation of the expression and prognostic significance of MMP14 and PKM2 in pancreatic cancer by tissue microarray

**Figure 9:**
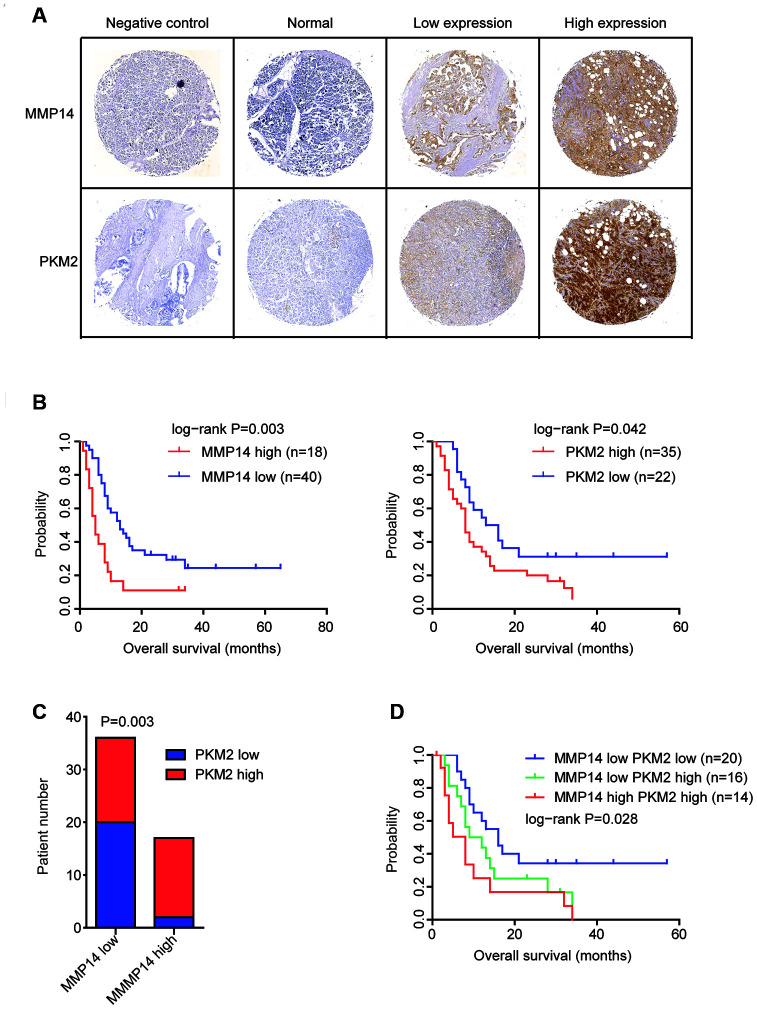
**Validation of the expression and prognostic significance of MMP14 and PKM2 in pancreatic cancer by tissue microarray.** (**A**) Representative photographs of immunohistochemical features of the stained sections of MMP14 and PKM2 in pancreatic cancer tissues and adjacent normal tissues. (**B**) Kaplan-Meier survival analysis was used to determine the different overall survival of pancreatic cancer patients with highly stained MMP14 or PKM2 (red) and pancreatic cancer patients with low stained MMP14 or PKM2 (blue). P values were generated from Log-rank test. (**C**) Contingency graphs showed the number of pancreatic cancer patients with both high MMP14 and PKM2 expression. P values were determined by Chi-square test. (**D**) Kaplan-Meier plotters demonstrated the different overall survival of pancreatic cancer patients with different expression levels of MMP14 and PKM2.

Further, using commercial tissue microarray, we determined the protein expression levels of MMP14 and PKM2 in Chinese pancreatic cancer patients. Totally, 60 pancreatic cancer tissues and adjacent normal tissues were tested. The representative immunohistochemical features of the stained sections of MMP14 and PKM2 were shown in Figure 9A. We found that compared with the adjacent normal tissues, MMP14 and PKM2 were strongly and positively stained in pancreatic cancer tissues (Figure 9A).

The protein expression levels of MMP14 and PKM2 in pancreatic cancer tissues were also varied significantly. We identified 18 pancreatic cancer tissues were with high MMP14 expression and 40 pancreatic cancer tissues were with low MMP14 expression (Figure 9A). Also, we found that 35 PKM2 highly expressed and 22 PKM2 lowly expressed pancreatic cancer tissues (Figure 9A). Consistent with the bad prognosis of MMP14 and PKM2, pancreatic cancer patients with higher expression of MMP14 or PKM2 had more unfavorable clinical overall survival (Figure 9B).

Previously, we demonstrated the positive correlations of MMP14 and PKM2 at mRNA expression levels in GSE71729, GSE78229 and TCGA datasets (Figure 8A). In the immunohistochemical features, we found that in the 18 pancreatic cancer tissues with highly stained sections of MMP14, 83% pancreatic cancer tissues were also with highly stained sections of PKM2 (Figure 9C). Those results further confirmed the correlation of MMP14 and PKM2 in pancreatic cancer. Furthermore, we showed that pancreatic cancer patients with both high MMP14 and PKM2 stained sections were with lowest overall survival (Figure 9D). All those results were consistent with our previous results derived from TCGA and GEO datasets.

## DISCUSSION

The development of pancreatic cancer is driven by KRAS, TP53, CDKN2A and SMAD4 genetic alterations through a particular sequence [28, 29]. KRAS mutation and CDKN2A deletion are occurred in the initiating stage of pancreatic cancer [30, 31]. In contrast, TP53 mutation and SMAD alteration are occurred in the later tumor progression [32], which promote the metastasis of pancreatic cancer cells [33]. Moreover, the invasive phenotypes of pancreatic cancer are required the coordination of KRAS, TP53 and SMAD alterations [22, 23]. Furthermore, study of 356 patients from Dana-Farber/Brigham and Women’s Cancer Center [24] and our results derived from the TCGA PAAD datasets showed that the KRAS, TP53 mutation and CDKN2A deletion were synergistically determined the clinical overall survival of patients with pancreatic cancer. Previously, SMAD4 gene mutation was reported to be associated with the poor prognosis of patients with pancreatic cancer [34]. However, our results suggested that SMAD4 alteration was not related to the overall survival of pancreatic cancer. Based on our results, molecular assessment of KRAS, TP53 and CDKN2A alterations may help guiding the prognosis of patients with pancreatic cancer.

Driver genetic alterations, like KRAS and TP53 mutation, may affect hundred genes to induce the transformation of normal cells into malignant cells. Using TCGA PAAD datasets, we identified 1575 genes commonly regulated by KRAS mutation, TP53 mutation and CDKN2A deletion in pancreatic cancer. Pancreatic cancer is a heterogeneous disease, and results derived from one cohort patients may not be repeated in other group of patients [35]. To avoid this inconsistence, we used 7 datasets to determine the prognostic effects of the commonly regulated genes and used 9 datasets to test the expression levels of the commonly regulated genes in normal and malignant pancreatic cancer tissues. Those integrated analysis revealed the prognostic significance of MMP14 and PKM2 in patients with pancreatic cancer. Previous results showed that MMP14 binding protein MTCBP-1 regulated the metastasis of pancreatic tumor cells [36] and PKM2 was over-expressed in pancreatic cancer [37]. However, the associations of MMP14, PKM2 and overall survival of pancreatic cancer patients were unclear. In present study, we showed that MMP14 and PKM2 were up-regulated by KRAS, TP53 mutation or CDKN2A deletion and the higher expression levels of MMP14 and PKM2 were associated with the worse prognosis. MMP14 combined with PKM2 could be used as prognostic marker in patients with pancreatic cancer.

Overall, by integrated analysis of TCGA and GEO datasets, our results provide deep understandings of how the KRAS, TP53, CDKN2A and SMAD4 genetic alterations and their related genes influence the clinical overall survival of pancreatic cancer patients. Our results also suggest new prognostic markers of MMP14 and PKM2 in pancreatic cancer. Furthermore, the prognostic effects of MMP14 and PKM2 are validated in Chinese pancreatic cancer patients. Although, the functions and correlations of MMP14 and PKM2 in pancreatic cancer patients require further elucidation, the combination of MMP14 and PKM2 could be used as better biomarkers to predict the overall survival of pancreatic cancer patients.

## MATERIALS AND METHODS

The TCGA Pancreatic adenocarcinoma (PAAD) gene expression, gene mutation and clinical information datasets were downloaded from TCGA hub (https://tcga.xenahubs.net). The gene expression series matrix of pancreatic cancer patients and clinical overall survival datasets were downloaded from the GEO website (http://www.ncbi.nlm.nih.gov/geo), including GSE21520, GSE28735, GSE57495, GSE71729, GSE78229 and GSE79668 datasets. The gene expression series matrix of pancreatic normal and malignant tissues was downloaded from GSE15471, GSE16515, GSE28735, GSE53452, GSE56560, GSE60646, GSE62452, GSE71729 and GSE71989 datasets.

### Oncoprints of KRAS mutation, TP53 mutation, CDKN2A deletion and SMAD alteration

The genomic tendency of KRAS mutation, TP53 mutation, CDKN2A deletion and SMAD alteration in patients with pancreatic cancer were downloaded from cbioportal (version 3.2.0, http://www.cbioportal.org/index.do) based on the TCGA PAAD datasets.

### KRAS mutation, TP53 mutation, CDKN2A deletion associated transcriptomic profiling

The KRAS mutation, TP53 mutation, CDKN2A deletion regulated genes were determined using paired Student’s t test based on TCGA RNA-seq datasets.

### GEO data processing

The GEO expression datasets were processed using R software (version 3.5.0, https://www.r-project.org/). Data splitting, applying and combining was applied by ‘plyr’ package (version 1.8.5), based on the constructions downloaded from bioconductor (https://cran.r-project.org/web/packages/plyr/index.html).

### Venn diagram

KRAS mutation, TP53 mutation, CDKN2A deletion commonly regulated genes were described by Venn diagrams which were generated from comparing lists tool VENNY 2.1 software (http://bioinfogp.cnb.csic.es/tools/venny/index.html).

### Kyoto Encyclopedia of Gens and Genomes (KEGG) signaling pathway enrichment analysis

The enriched KEGG signaling pathways were determined using The Database for Annotation, Visualization and Integrated Discovery (DAVID) website (version 6.8; https://david.ncifcrf.gov). Enrichment results with P-value < 0.05 were considered to be statistically significant.

### Heatmap presentation

The heatmaps were generated by ‘pheatmap’ package (version 1.0.12) using R software. The basic usage of ‘pheatmap’ was downloaded from bioconductor (https://cran.r-project.org/web/packages/pheatmap/). The ‘average’ method was used to determine the clustering scale. The ‘correlation’ method was used to determine the clustering distance.

### Survival analysis

Kaplan-Meier estimator was generated by ‘survival’ package (version 3.1-8) in R statistics software to reveal the prognostic effects of the genomic alterations and MMP14 and PKM2 expression. The “survival” package and the basic usage were downloaded from bioconductor. P values were determined using Log-rank test.

### Univariate and multivariate cox regression

Univariate and multivariate cox regression analysis were carried out by R software “survival” package. Log-rank test was used to calculate the P values.

### Spearman correlation

Spearman correlation was determined by “lm” method in R software to study the correlation between MMP14 and PKM2 expression in patients with pancreatic cancer.

### Immunohistochemistry

The protein expression levels of MMP14 and PKM2 in Chinese pancreatic cancer tissues were detected by immunohistochemistry using commercial tissue microarray (HPanA120Su02, Shanghai OUTDO Biotech). 60 pancreatic cancer tissues and 60 corresponding adjacent normal tissues were tested. Rabbit anti-MMP14 antibody (PA1-38193, ThermoFisher) and rabbit anti-PKM2 antibody (D78A4, Cell Signaling Technology) were used as primary antibodies. Normal rabbit immunoglobulin G (Abcam) was used as a negative control. The protocol of immunohistochemistry was previously described [38]. The stained intensity of MMP14 and PKM2 was determined by three pathologists from Shanghai Changhai Hospital in a blinded manner.

### Statistical analysis

The box plots were generated from GraphPad Prism software (version 5.0). Statistical analysis was performed using the paired Student’s t test using R software. P value less than 0.05 was chosen to be significantly different.
